# Hidden threats: exploring biofilm communities in broiler houses and pig nursery units drinking water lines

**DOI:** 10.1186/s12866-026-04790-6

**Published:** 2026-03-05

**Authors:** Ulric Van Rossum, Marc Heyndrickx, Geertrui Rasschaert, Niels Demaître, Faizan Ahmed Sadiq, Nico Boon, An Cools, Koen De Reu

**Affiliations:** 1 Fisheries and Food - Technology and Food Unit, ILVO - Flanders Research Institute for Agriculture, Melle, Belgium; 2 Inagro, Rumbeke-Beitem, Belgium; 3 Evap Proefbedrijf Pluimveehouderij, Geel, Belgium; 4 CMET – Center for Microbial Ecology and Technology, Faculty of Bioscience Engineering, Ghent University, Gent, Belgium; 5 Faculty of Veterinary Medicine, Ghent University, Gent, Belgium; 6 Advanced Therapies Group, School of Dentistry, Cardiff University, Cardiff, Wales CF14 4XY UK

**Keywords:** Biofilm, Broiler, Piglets, Drinking water systems, Microbiota, *Staphylococcus saprophyticus*

## Abstract

**Background:**

Drinking water systems (DWS) are often an overlooked source of microbial contamination of drinking water in broiler and piglet production. Persistent biofilms within water lines can act as reservoirs of contamination, reintroducing microorganisms into the flowing water and potentially compromising animal health. This study investigates the microbial composition of biofilms in the DWS of broiler houses and pig nursery units, their impact on drinking water quality, and the influence of the source water on both water quality and biofilm communities.

**Results:**

The bacterial load of DWS biofilm swabs, collected at the end of production cycles before cleaning and disinfection was evaluated, and the dominant bacterial taxa were identified. Furthermore, 16S gene metabarcoding was applied to the biofilm samples. No significant differences in microbial load were observed between the two sectors, with a median total aerobic count of 3.6 log CFU/cm^2^. Enterococci, a faecal indicator, were detected in 80% of all samples. Moreover, *Escherichia coli* was found more frequently in broiler houses (47%) than in pig nursery units (27%). The two dominant identified genera were *Staphylococcus* and *Pseudomonas*. The *Staphylococcus saprophyticus* species was the most frequently identified isolate, accounting for 10.6% of all isolates across both broiler houses and pig nursery units. In broiler houses, the next most frequently identified species were *Pseudomonas aeruginosa* (5.6%) and *Stenotrophomonas maltophilia* (5.3%). In contrast, in pig nursery units, *Pseudomonas fluorescens* (6.3%) and *Psychrobacter faecalis/pulmonis* (5.5%) were most frequently identified. Research showed that the drinking water microbial community not only depended on the source water but was also influenced by biofilms in DWS, as similar bacterial taxa were found in both the drinking water at the drinking nipples and in biofilms on water-contact surfaces.

**Conclusions:**

The presence of faecal indicator bacteria and potential animal pathogens underscores the risks associated with the biofilms. These biofilms can contaminate drinking water to animals, underscoring the need for targeted strategies to monitor and mitigate biofilm formation.

**Supplementary Information:**

The online version contains supplementary material available at 10.1186/s12866-026-04790-6.

## Introduction

Ensuring high-quality drinking water is crucial for the overall health, welfare, productivity of pigs and poultry and for protecting human health through the safety of animal-derived food products [1, 2]. Water quality is determined by both its chemical and microbiological composition. However, the presence of pathogenic bacteria in drinking water remains a significant challenge. In broiler houses and piglet nursery units, *Escherichia coli*, *Campylobacter* spp., *Salmonella* spp., *Pseudomonas aeruginosa*, enterococci, and streptococci are frequently detected [3–9]. To monitor microbiological water quality, analyses include microbiological indicators recommended by the Belgian Animal Health Service, such as total aerobic counts, total coliforms, *E. coli*, enterococci, *Clostridium perfringens*, and counts of yeasts, and moulds [10, 11].

An important determinant of drinking water quality is the formation of microbial biofilms on water-contact surfaces in drinking water systems (DWS), which serve as major reservoirs of pathogenic and other harmful bacteria [12, 13]. Conditions in DWS, including stable temperatures of 25 °C or above, the presence of organic matter, and variable microbiological composition and loads from different source waters, create an ideal environment for biofilm proliferation [14–16]. Biofilms are sessile microbial communities encased in an extracellular polymeric substance (EPS) matrix [17]. This matrix not only facilitates surface adhesion but also offers protection against environmental stress conditions, including cleaning procedures and disinfectants [18–21]. Bacteria commonly dominating biofilms in DWS used for animal production include *Pseudomonas* spp., *Sphingomonas* spp., *Acinetobacter* spp., and *Aeromonas* spp. [22–25].

Water quality can be managed through water treatment and various cleaning and disinfection (C&D) procedures on the DWS. Farmers typically use oxidisers and acids for cleaning, often between production rounds during vacancy [16, 25, 26]. The European Chemical Agency also permits the use of low-concentration disinfectants in drinking water during production rounds [27]. However, a study showed that these treatments often fail to eliminate biofilms, resulting in persistent bacterial contamination in broiler house water systems [25]. Beyond water contamination, persistent biofilms pose additional challenges by interfering with medication and supplement administration, as they can sequester active compounds and cause underdosing in animals [28]. Moreover, when bacteria within a biofilm develop resistance to a specific antimicrobial agent or environmental stress, they may also exhibit cross-resistance to other agents or stresses to which they have not been exposed [29, 30]. Additionally, biofilms also enhance horizontal gene transfer and intercellular communication, further increasing the spread and persistence of antimicrobial resistance [13, 31].

Despite the already available research into microbial contamination of drinking water systems in animal production, knowledge gaps remain regarding biofilms in broiler houses and pig nursery units, particularly in terms of the microbiological composition and its impact on drinking water quality. Therefore, this study aimed to analyse the presence and microbiological composition of biofilms in DWS within broiler houses and pig nursery units and assess how these biofilms influence the microbial quality and composition of the drinking water. These objectives were addressed by analysing biofilm samples from DWS and drinking water samples collected from 15 broiler houses and 15 pig nursery units, on which microbiological plate enumeration and identification were performed. The study also investigated differences between both sectors (poultry and pig) and the impact of current cleaning and disinfection procedures and source waters on biofilm-associated microbial communities.

## Materials and methods

### Sample collection

Biofilm samples from 15 broiler houses and 15 pig nursery units were gathered from DWS in Belgium between December 2022 and January 2024. Source waters, water treatments and disinfections performed during production in those premises are listed in Table 1. Samples were taken from the inner surface of the pipelines either after removing the drinking nipples or at the end of the pipeline after removing the tap. Sampling occurred at the end of an animal production round, after the animals had been removed and before any C&D of the DWS had taken place. Utilizing premoistened sterile FLOQSwabs® (Copan Diagnostics, California USA, 520 C) with 2 ml Dey-Engley Neutralizing buffer (Sigma-Aldrich, Diegem Belgium, D3435), biofilm samples were collected by thoroughly swabbing a 20 cm^2^ area surface after draining the pipes [25]. In parallel, drinking water samples were collected from both the source water and the drinking nipple before any C&D treatment. The sampling followed standard procedures (ISO 8199 [32] and 19458 [33]). Afterwards, all samples were transported to the laboratory in a cooler with ice packs and maintained at 3 ± 2 °C until analysis. Analysis were performed within 24 h of collection.

**Table 1:** Source water, treatment, and disinfection methods used in broiler houses and pig nursery units

		Broiler houses (n)	Pig nursery units (n)
Type of source water	Ground	8	6
	Surface	1	2
	Rain	1	3
	Tap	5	4
Water treatment^a^	Demineralization	1	1
	Acidification	2	5
	De-ironing	1	2
	Filtration	2	7
	Ultrafiltration	1	1
	Ultrasonic	1	1
	None	10	4
Active compound of water disinfection during production	UV	2	4
	Hydrogen peroxide (0.004%−0.010%^b^)	6	8
	Chlorine (0.001%−0.050%)	1	1
	Chlorine dioxide (0.0005%−0.0150%)	1	3
	None	7	3
Active compound of DWS disinfection during vacancy	Hydrogen peroxide (0.01%−4.00%)	4	6
	Chlorine (2.00%)	2	1
	Chlorine dioxide (0.03%−0.05%)	1	4
	Potassium peroxymonosulfate (1.0%)	2	n.a
	Peracetic acid + hydrogen peroxide (1.0%)	1	n.a
	None	5	4

*n.a* = Not applicable, *n* = Number of farms

^a^Applied before usage

^b^Range of applied concentration of the disinfection product (v/v)

### Microbiological characterisation

#### Microbiological enumerations, detection and environmental parameters

The inoculum was prepared by adding 8 ml of Dey-Engley Neutralizing buffer (Sigma-Aldrich, Diegem Belgium, D3435) to the swabs containing biofilm material. Appropriate serial dilutions were then made in Buffered Peptone Water (BPW; BioTrading, Berlin Germany, K168B009AA), and either spiral-plated or spread-plated onto the corresponding selective or non-selective culture media. Microbial populations were enumerated according to ISO standards for microbiological water quality assessment. Specifically, total aerobic count (TAC) at 21 °C and 37 °C, coliforms, enterococci, *Pseudomonas* spp., *C. perfringens*, yeasts and moulds (ISO 6222 [34], ISO 9308 [35], ISO 7899 [36], ISO 13720 [37], ISO 14189 [38], ISO 21527 [39]) were enumerated, as briefly described in Table 2. Plates were incubated at 21 °C and 37 °C to count both environmental and warm-blooded-animal–associated microorganisms [40]. *C. perfringens* colonies were enumerated without performing the acid phosphatase biochemical confirmation test; instead, identification was confirmed using Matrix-Assisted Laser Desorption/Ionization Time-of-Flight Mass Spectrometry (MALDI-TOF). Furthermore, detection of *Escherichia coli* was performed by adding 1 ml of the inoculum to 9 ml of BPW and incubating at 37 °C for 24 h. The resulting enriched solution was inoculated with 1 ml on Rapid’*E.coli* 2 medium (Bio-Rad, Temse Belgium, 3,564,024) and incubated for 24 h at 37 °C (ISO 9308). Furthermore, both types of drinking water samples were analysed for TAC (22 °C and 36 °C), coliforms, *E. coli*, enterococci, *C. perfringens*, yeast and moulds, following the same ISO standards and methods used for the swab samples. Environmental conditions (pH, temperature, total oxidized nitrogen, total orthophosphate, total hardness and conductivity were measured for every sample following standard procedures (ISO 10523 [41], ISO 13395 [42], ISO 10304–1 [43], ISO 6059 [44], ISO 7888 [45]).

**Table 2:** Culture media used for the enumeration of the described microbial groups from the biofilm samples

	Incubation				
Microbial Group	T (°C)	Time	Culture Medium	Supplements	Lower enumeration limit (log CFU/cm^2^)
TAC	21	72 h	Plate Count Agar (Oxoid, Hants United-Kingdom, CM0325)	n.a	1.7
TAC	37	48 h	Plate Count Agar (Oxoid, CM0325)	n.a	1.7
Coliforms	37	24 h	Rapid'*E.coli* 2 (Bio-Rad, 3,564,024)	n.a	0.7
Enterococci	44	24 h	Slanetz and Bartley Agar (S&B; Oxoid, CM0377)	n.a	0.7
*C. perfringens*	44	24 h	Perfringens Agar Base (Oxoid, CM0587)	Tryptose Sulphite Cycloserine (Oxoid, SR0088)	−0.3
Yeasts and moulds	25	5 days	Yeast Extract Glucose Chloramphenicol Agar (Becton Dickinson, 219,001)	n.a	0.7
*Pseudomonas* spp.	30	48 h	Pseudomonas Agar Base (Oxoid, CM0559)	CFC Selective Supplement (Oxoid, SR0103)	0.7

*n.a.* = Not applicable

#### Statistical analysis

Of the 200 biofilm samples (98 from broiler houses and 102 from pig nursery units) and 60 drinking water samples (15 from the source water and 15 from drinking nipple water in each sector), only countable samples were used for the statistical analysis, which was conducted using GraphPad Prism 10.1. The enumerations were log-transformed and described in CFU/ml or CFU/100 ml for drinking water counts and in CFU/cm^2^ for the swabs. Significant differences in counts between the broiler house samples and the pig nursery units were analysed using the non-parametric Mann–Whitney U test with Holm–Šidák correction for multiple comparisons (*p* < 0.05) for each counted microbial group. Furthermore, the Spearman correlation of TAC (22 °C and 36 °C) between source water and drinking nipple water samples and between water samples and biofilm samples was calculated across both sectors and all farmhouses. The Spearman correlation was also assessed between all measured environmental parameters and the TAC for drinking water and biofilm samples. Significant results were reported at *p*-values < 0.05.

#### Collection of microbial isolates

A total of 1284 isolates representing the dominant microbiota from the biofilm samples across all agar media was collected. Dominant microbiota selection was based on morphological variation on the agar media, representing the highest decimal dilution with bacterial growth. From each plate, one to ten colonies were chosen, depending on the degree of morphological variation observed. For drinking water samples, dominant microbiota selection was performed only using the TAC (21 °C and 37 °C) plates taken at the source water and the drinking nipple water. Colonies were picked, streaked and incubated on Plate Count Agar (Oxoid, CM0325) media plates at least three times to obtain pure cultures. Pure cultures from TAC, coliforms, and *Pseudomonas* spp. were incubated in 1 ml Brain Heart Infusion Broth (BHI; Oxoid, CM1135) supplemented with 15% glycerol (Fisher Scientific, Fair Lawn New Jersey, AC332030100) at appropriate temperatures for 24 h. Colonies originating from Slanetz and Bartley agar (Oxoid, CM0377) were cultured in M17 Broth (Becton Dickinson, 218,561) and *C. perfringens* colonies in Reinforced Clostridial Broth (Oxoid, CM0149), all supplemented with 15% glycerol. All isolates were stored at −70 °C.

#### Bacterial isolate identification through MALDI-TOF MS

The isolates were analysed using the bench-top microflexTM LRF mass spectrometer (Bruker Daltonics, Kontich Belgium). The fuzzy-logic-based AutoXecute™ within the flexControlTM 3.4 module and the Bruker Maldi Biotyper (MBT) 4.1 from the integrated CompassTM software were employed for mass spectrometer control and for comparison with the MBT Compass reference library 2022 (Bruker Daltonics) to identify the isolates. Isolates with scores between 1.70 and 1.99 were assigned to the putative genus level, whereas isolates with scores ≥ 2.00 were identified at the species level. Samples were prepared following the formic acid extraction protocol as described by Bruker Daltonics. Initially, direct protein extraction was performed on fresh colonies for spectral acquisition and identification. Isolates that could not be identified at the species level underwent an indirect protein extraction for identification. A Bacterial Test Standard of *E. coli* provided by the manufacturer (Bruker Daltonics, 8,255,343) was included in every run for calibration purposes.

#### Bacterial isolate identification by 16S rRNA sequencing

Bacterial isolates not identified at the putative species level by MALDI-TOF MS were further identified through 16S rRNA sequencing. First, crude cell bacterial lysates were prepared by washing pure cultures in 1 ml of Ringer Solution (Oxoid, BR0052G), and the pellet was suspended in 50 µl of 0.1 M NaOH and 50 µl of 0.25% (v/v) Sodium Dodecyl Sulphate (SDS; Sigma-Aldrich, L3771). The solution was then heated for 17 min at 90 °C. Subsequently, 1 µl of the crude cell lysate was used as the template for PCR. The 16S rRNA gene was targeted for amplification using the primers 16F27-1 (pA, 5′−3′sequence: AGA GTT TGATCC TGG CTC AG) and 16R1522 (pH, 5′−3′sequence: AAG GAG GTG ATC CAG CCG CA), yielding an amplicon of approximately 1500 bp [46]. The PCR was performed as previously described [25]. Subsequently, the PCR product was subjected to Sanger sequencing using forward and reverse primers by GENEWIZ from Azenta Life Sciences (Leipzig, Germany). Sequences exceeding 500 bp were analysed using BLASTN® [47] against the 16S RefSeq Targeted Loci rRNA database [48] using the Geneious Prime 2023 bioinformatics software. Alignments with sequences from the database exhibiting the highest pairwise identity score (minimum 98%) were used to identify isolates at the putative species or species group level.

#### Metabarcoding of biofilm samples

Metabarcoding analysis was conducted on 51 biofilm samples (one or two per farm), including 27 from 15 broiler houses and 24 from 13 pig nursery units. A pellet was obtained from 2 ml of the mother biofilm suspension and washed with 1 ml of a solution containing 0.15 M NaCl and 0.015 M trisodium citrate. DNA was then extracted as previously described [49, 50]. The obtained DNA was suspended in 50 µl of nuclease-free water (Qiagen, Venlo, The Netherlands, 129,114) and finally stored at −25 °C until analysis. The genomic DNA samples were sent for 16S metagenomic sequencing through Illumina technology by Macrogen Europe (Amsterdam, The Netherlands). The amplicon sequencing dataset was pre-processed in the Ubuntu operating system. Removal of adapters at the 3' end, removal of primers and quality filtering were performed with the command-line tool cutadapt 3.0 [51]. Merging of forward and reverse reads was performed with paired end read merger tool 0.9.8 (PEAR [52],). Filtering and taxonomy assignments were performed following the DADA2 pipeline with R studio 4.4.1. [53]. Bacterial alpha diversity was assessed using the Shannon and Simpson (Gini) indices via the phyloseq R package [54]. Diversity indices were analysed across farm types, source waters, and the use of DWS disinfection during vacancy periods. The Shapiro–Wilk test was applied to evaluate the normality of richness distributions. As the data did not meet normality assumptions, the non-parametric Kruskal–Wallis test was conducted with a significance threshold of *p* < 0.05. To investigate overall bacterial community composition, beta-diversity was analysed at the amplicon sequence variant (ASV) level using Bray–Curtis dissimilarities of relative abundance data. A Non-metric Multidimensional Scaling (NMDS) and Principal Coordinates Analysis (PCoA) were performed using phyloseq. Differences in microbial community diversity at the genus level were evaluated using Permutational Multivariate Analysis of Variance (PERMANOVA) based on Bray–Curtis dissimilarity, using the vegan R package [55]. Before conducting PERMANOVA, the assumption of homogeneity of multivariate dispersion was assessed, and PERMANOVA was only performed when this assumption was met (999 permutations).

## Results

### Microbiological enumerations

#### DWS biofilm surface samples

**Fig. 1:**
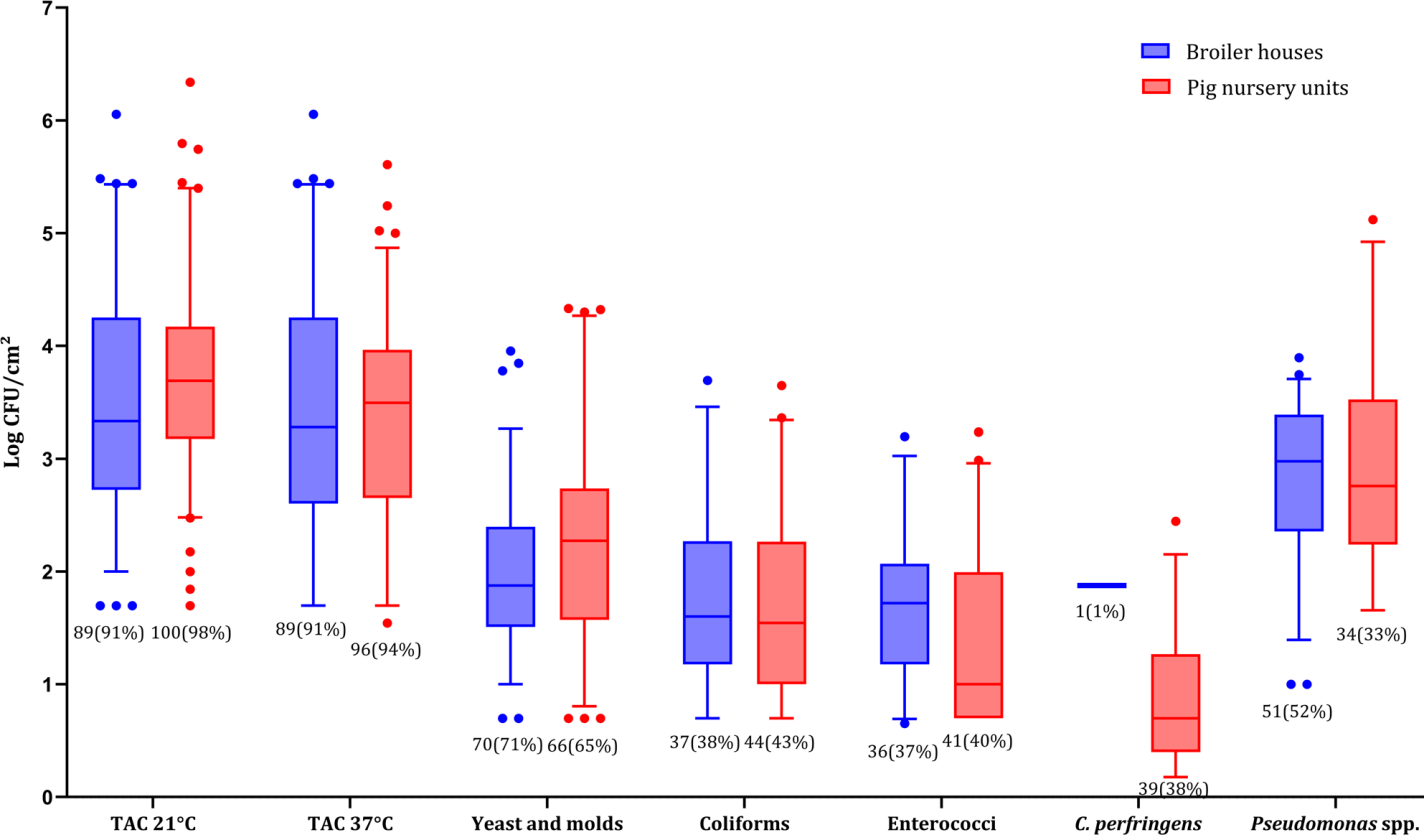
Values for total aerobic count (TAC) at 21°C and 37°C, yeast and moulds, coliforms, enterococci, *C. perfringens*, *Pseudomonas *spp. (log CFU/cm²) enumerations from biofilm samples collected from the surface of drinking water systems within broiler houses and pig nursery units. In each boxplot, the horizontal line crossing the box represents the median, the bottom and top of the box represent the lower and upper quartiles, the vertical top line represents the upper interquartile range, and the vertical bottom line represents the lower interquartile range. The values and percentages under each box represent the number of countable samples

A total of 200 biofilm (98 from broiler houses and 102 from pig nursery units) swab samples were collected from 15 broiler houses and 15 pig nursery units from which microbiological enumeration was assessed (Fig. 1). The median TAC of countable samples per farm ranged from 2.8 and 5.6 log CFU/cm^2^ at 21 °C and from 2.3 to 5.3 log CFU/cm^2^ at 37 °C across both farm sectors. Coliforms, enterococci, yeast and moulds, and *Pseudomonas* spp. were countable in 41%, 39%, 68%, and 43% of the samples, with counts ranging from 0.7 to 3.4 log CFU/cm^2^, 0.7 to 2.3 log CFU/cm^2^, 1.1 to 4.0 log CFU/cm^2^, and 2.2 to 4.5 log CFU/cm^2^, respectively. *C. perfringens* was only detected once in broiler houses, while it was detected in 38% of the pig nursery units’ samples, with median farm-level counts ranging from 0.2 to 2.5 log CFU/cm^2^. Finally, *E. coli* was detected in 12% of broiler house samples and 7% of pig nursery units. No significant differences (*p* < 0.05) were observed between the microbial parameters of the broiler house samples and those from the pig nursery units. The counts at each farmhouse are available in Table S1.

#### Drinking water samples

**Fig. 2:**
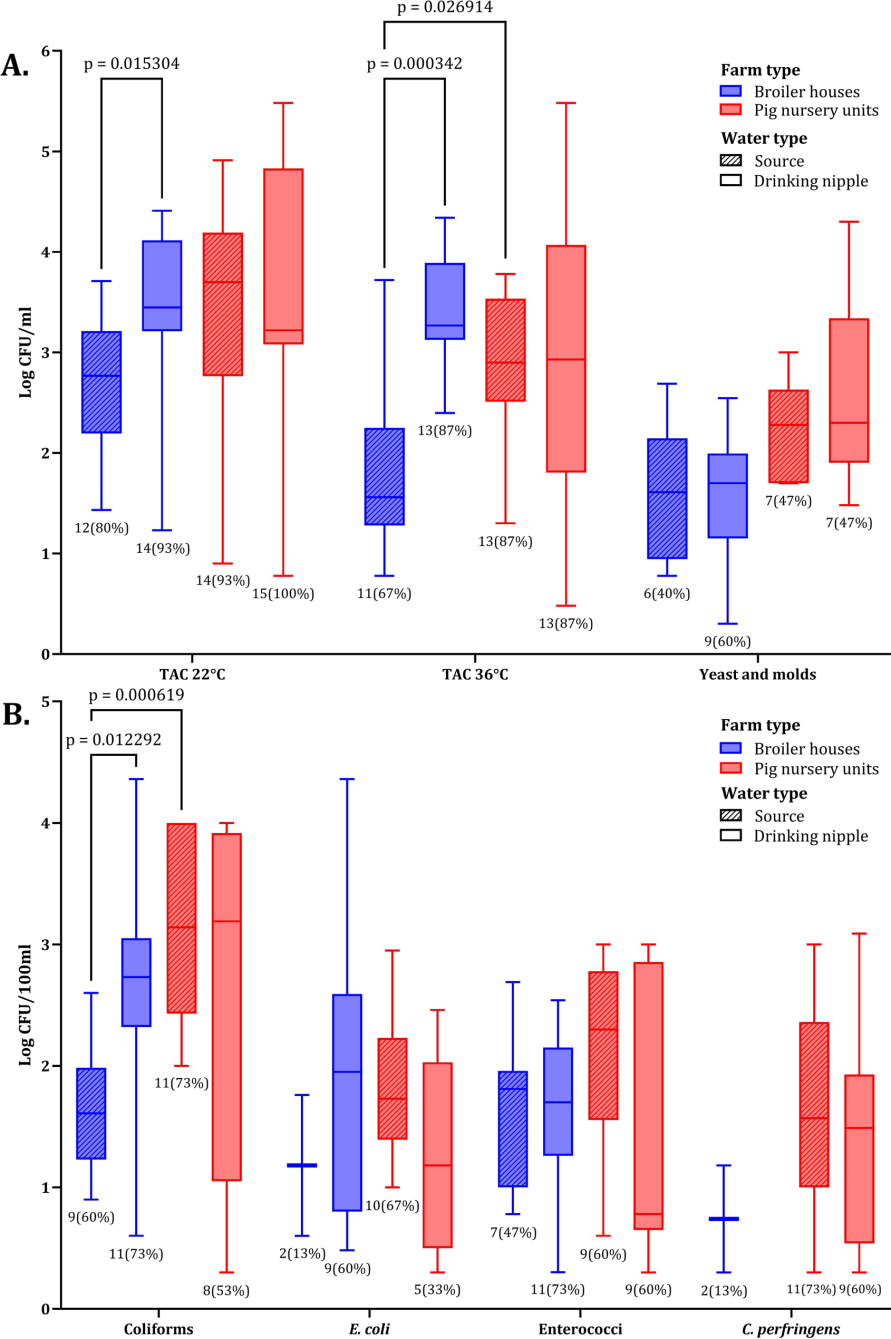
Values for total aerobic count (TAC) at 22 °C and 36 °C, yeast and moulds enumerations (log CFU/ml, **A** from drinking water samples collected at the source and the drinking nipples of drinking water systems within broiler houses and pig nursery units. Values for coliforms, *E. coli,* enterococci, and *C. perfringens* (log CFU/100 ml) (**B**). In each boxplot, the horizontal line crossing the box represents the median, the bottom and top of the box represent the lower and upper quartiles, the vertical top line represents the upper interquartile range, and the vertical bottom line represents the lower interquartile range. The values and percentages under each box represent the number of countable samples. The p-values of significant differences between the source and end of pipeline enumeration boxes are shown according to the Mann–Whitney test at *p* < 0.05

In addition to the biofilm swab samples, drinking water samples were collected at each farm from the source water and the drinking nipple water in both sectors, and similar microbiological enumerations were performed on them. In broiler houses, the median microbial counts in source water were as follows: TAC at 22 °C and 36 °C were 2.8 and 1.6 log CFU/ml, respectively, while yeasts and moulds were detected at 1.6 log CFU/ml. Coliforms, *E. coli*, enterococci, and *C. perfringens* were counted at median levels of 1.6, 1.2, 1.8, and 0.7 log CFU/100 ml, respectively. The TAC median levels at 22 °C and 36 °C of the water samples collected from drinking nipples were 3.5 and 3.3 log CFU/ml, respectively, and yeasts and moulds remained at median levels of 1.6 log CFU/ml. Coliforms, *E. col*i and enterococci were detected at 2.7, 2.0, and 1.7 log CFU/100 ml, respectively, whereas, *C. perfringens* counts were below the enumeration limit. Statistical analysis revealed significant differences for TAC at 22 °C and 36 °C and coliform counts between source water and drinking nipple water samples. Additionally, all parameters, except for *C. perfringens*, were more frequently counted in drinking nipple water samples (Fig. 2). This indicates an increased microbiological load at the drinking nipples.

In pig nursery units, the median levels of the microbial counts in the source water were as follows: TAC at 22 °C and 36 °C were 3.7 and 2.9 log CFU/ml, respectively, while the yeast and mould median level was 2.3 log CFU/ml. Coliforms, *E. coli*, enterococci, and *C. perfringens* were present at 3.1, 1.7, 2.3, and 1.6 log CFU/100 ml, respectively. Water samples collected from drinking nipples showed the following median levels: TAC at 22 °C and 36 °C were 3.2 and 2.9 log CFU/ml, respectively, with yeast and moulds at 2.3 log CFU/ml. Coliforms, *E. coli*, enterococci, and *C. perfringens* were found at 3.2, 1.2, 0.8, and 1.5 log CFU/100 ml, respectively. The analysis showed no significant differences in the overall microbial parameters between the source water and the drinking nipple water samples (Fig. 2). Comparing both sectors, source water in broiler houses exhibited a lower microbiological load than water from pig nursery units. However, only a significant difference was observed in TAC at 36 °C (*p* = 0.026914) and coliform (*p* = 0.000619) counts. The drinking nipple water samples from both sectors showed comparable microbial counts, with no significant differences.

#### Microbial correlations across sample types

The TAC at 22 °C in the source water was significantly correlated with the TAC in the drinking water at the nipples across both sectors. Results on TAC at 36 °C showed no significant correlation between source water and drinking nipple water samples (Table S3). Moreover, the TAC at 37 °C of the biofilm samples was correlated with the drinking nipple water enumerations. No significant correlation was observed between the TAC counts of biofilm samples and source water samples, nor between biofilm samples and drinking nipple water samples at 21 °C (Table S3).

#### Other water measurements

The values of pH, temperature, total oxidised nitrogen, total orthophosphate, total hardness, and electrical conductivity were comparable between the source water and the drinking nipple water (Table S2). At 22 °C, the TAC of the drinking nipple water samples was correlated with conductivity, whereas at 36 °C, it showed correlations with pH and total hardness. Furthermore, the TAC at 37 °C in the biofilm swabs was correlated with pH and correlated with total hardness. No other significant correlations were found between TAC results in biofilm or drinking water samples and the measured parameters (Table S4).

### Identification of microorganisms in biofilm surface samples

#### Isolates originating from PCA

**Fig. 3:**
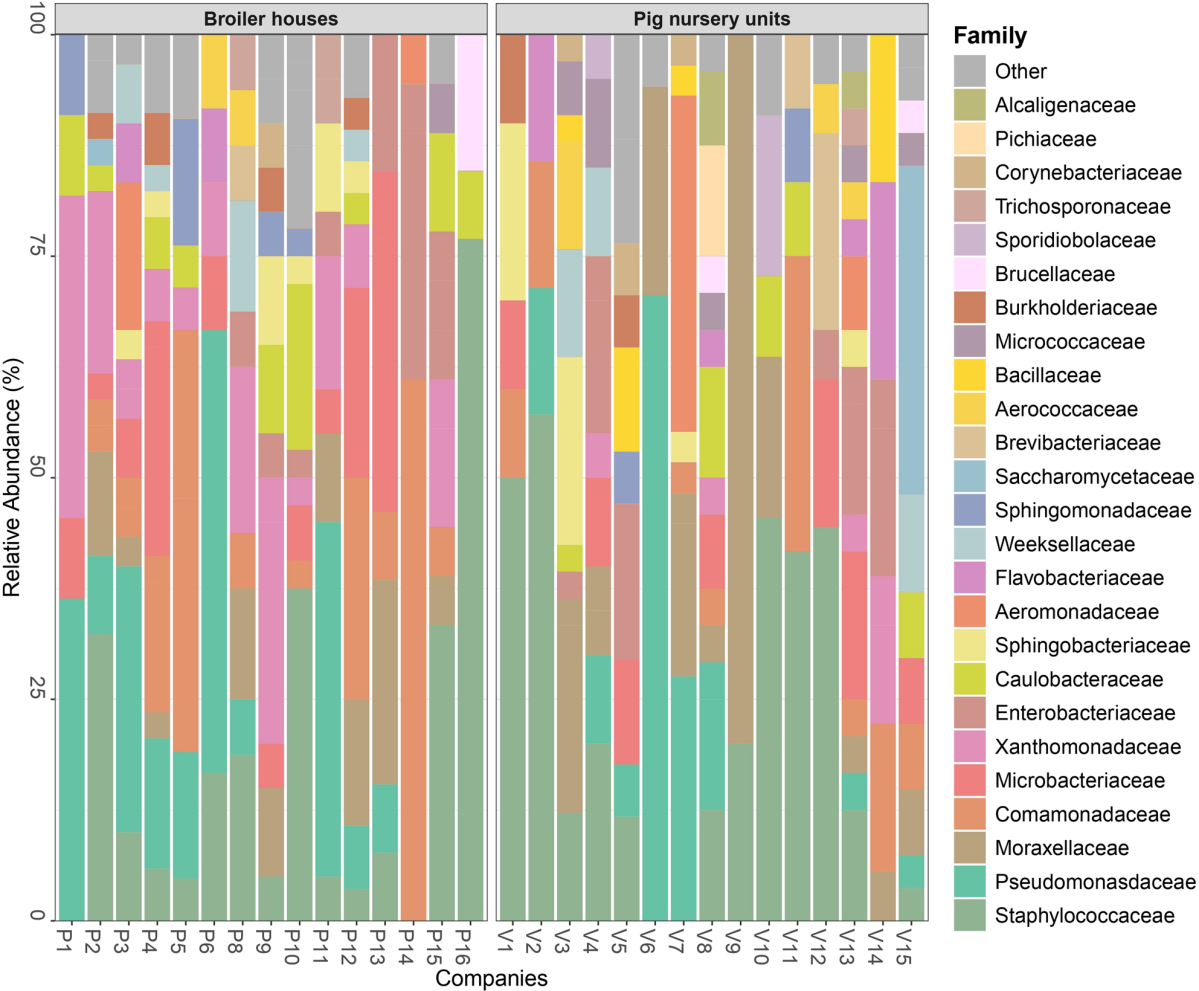
Cumulated histograms representing the relative abundance of the dominant taxa, cultivable on PCA at family level (top 25). Isolates originated from broiler houses (P1 to P15) and pig nursery units (V1 to V15) drinking water systems biofilm samples that were identified through MALDI-TOF (*n* = 464) and 16S rRNA (*n* = 128) sequencing

**Fig. 4:**
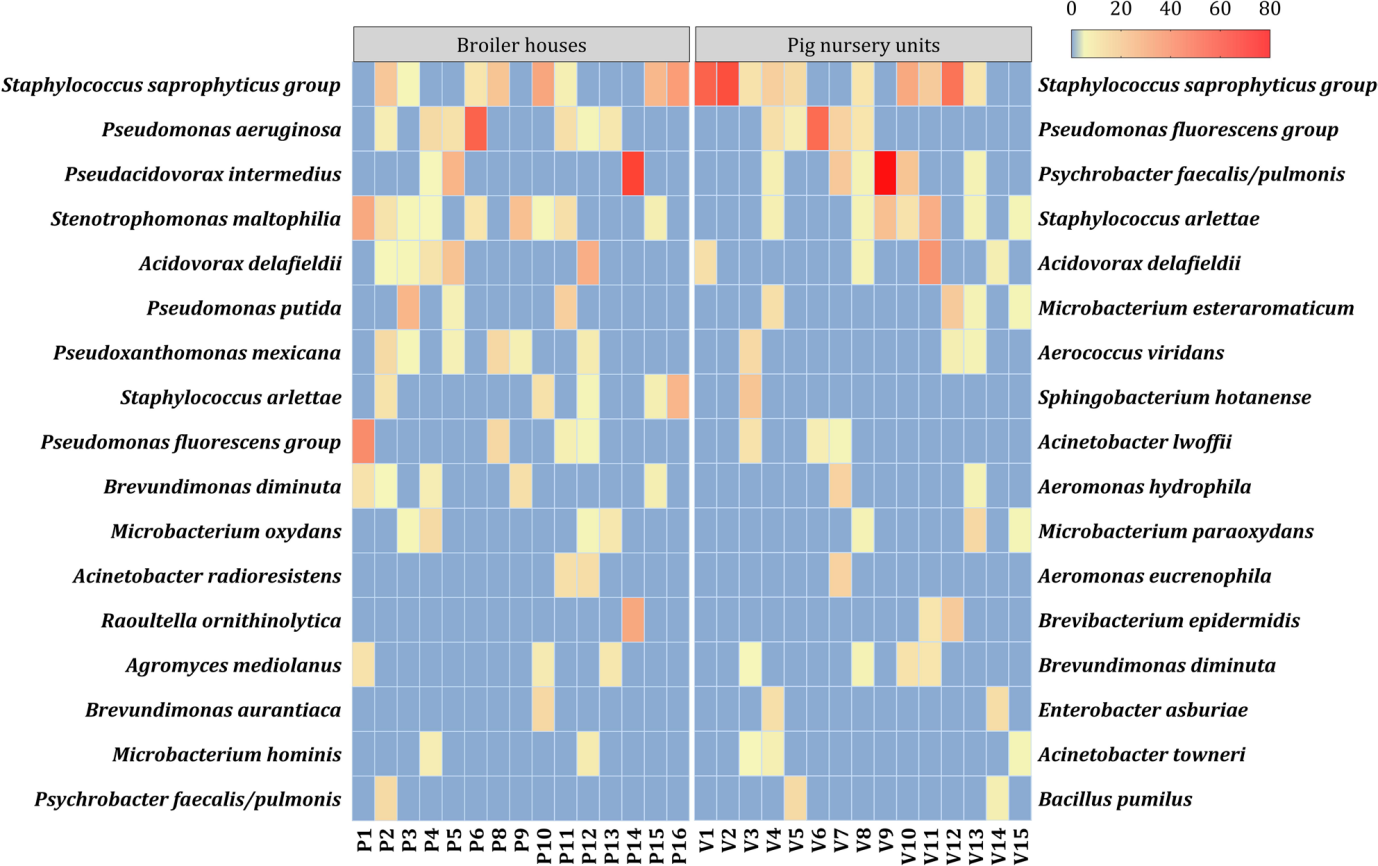
Heatmap of species composition. Heatmap showing the top 17 most abundant species retrieved from PCA. Isolates originated from broiler houses (P1 to P15) and pig nursery units (V1 to V15) drinking water systems biofilm samples that were identified through MALDI-TOF and 16S rRNA sequencing. The colour code indicates relative abundance, ranging from blue (low or no abundance) to yellow and to red (high abundance)

Dominant microbiota in biofilm samples collected from the inner surfaces of the water pipelines were identified using isolates grown on PCA culture media. In total, 592 isolates were further identified, from which 464 isolates with MALDI-TOF and 128 isolates with 16S RNA sequencing. The dominant phyla in the broiler and pig nursery sectors were Proteobacteria (61.9% and 48.7%, respectively), Firmicutes (18.1% and 21.6%), Actinobacteria (10.3% and 11.7%), and Bacteroidota (5.6% and 9.9%). Closely related species belonging to the *Staphylococcus saprophyticus* group, the *Pseudomonas aeruginosa* group and the *Pseudomonas fluorescens* group were grouped together as described in previous research [56–59]. The two most abundant families identified were for the broiler sector and pig nursery sector, respectively, *Staphylococcaceae* (16.5%, 16.8%) and *Pseudomonadaceae* (13.1%, 11.0%, Fig. 3). Within the *Staphylococcaceae* family, species from the *S. saprophyticus* group (*S. saprophyticus*, *Staphylococcus xylosus*, *Staphylococcus equorum*, and *Staphylococcus cohnii*) were most common, representing 10.1% for the broiler sector and 12.5% for the pig nursery sector (Fig. 4). The *Pseudomonadaceae* family was mainly represented by the *P. aeruginosa* group for the broiler sector and by the *P. fluorescens* group for the pig nursery sector, with prevalences of, respectively, 5.6% and 6.3%. Other notable families included *Comamonadaceae* (13.0% in the broiler sector, 5.5% in the pig sector), *Moraxellaceae* (6.3%, 11.4%), *Microbacteriaceae* (8.4%, 5.7%), and *Xanthomonadaceae* (11.6%, 2.2%; Fig. 3). Frequently identified species within these families were *Acidovorax delafieldii* (5.0%, 2.6%), *Psychrobacter faecalis/pulmonis* (1.3%, 5.5%), two closely related species [56], *Microbacterium esteraromaticum* (0.9%, 2.6%), and *Stenotrophomonas maltophilia* (5.3%, 1.1%, Fig. 4). In summary, the identified families and species mentioned above accounted for 61.9% and 30.2% of all isolates.

#### Isolates originating from PAB, RAPID’E.coli 2 and S&B

Microbial identification was also carried out from the isolates originating from the selective culture media. The predominant pseudomonads in the broiler house microbial communities were *Pseudomonas putida* (16%) and *P. fluorescens* (14%), both identified in 5 sampled farmhouses. *E. coli* (19%) was the most common coliform, identified in 6 farmhouses. The most prevalent enterococci were *Enterococcus casseliflavus* (14%) and *Enterococcus faecium* (14%), identified in 2 and 3 farmhouses, respectively (Table S5).

In the pig nursery units, the *P. fluorescens* (52%) species dominated the pseudomonads identified in 3 farmhouses. The primary coliform was *E. coli* (16%), identified in 4 farmhouses. *Enterococcus avium* (21%), found in 2 farmhouses, was the most frequently identified enterococcus. In both sectors, *S. arlettae* (18% in broiler houses and 50% in pig nursery units) and *S. saprophyticus* (14% for both) were commonly identified on S&B medium (Table S5).

### Microorganism identification present in drinking water samples from PCA

The dominant microbial microbiota in drinking water samples collected from both the source water and the drinking nipple water was identified using isolates grown on PCA culture media. Of the 166 isolates, 59 did not grow after purification. At the source in the broiler house drinking water, species from the *Pseudomonas* (24%) and *Aeromonas* (12%) genera, including *P. fluorescens* and *Aeromonas veronii*, were most common and found in 7 and 2 farmhouses. At the end of the pipeline, *S. maltophilia* (20%) and *P. aeruginosa* (14%) were the dominant species, present in 3 and 4 broiler houses (Table S6). Within pig nursery units, the species found in both the source water and the drinking nipple water included *S. arlettae*, *P. fluorescens*, *Janthinobacterium lividum, Aeromonas bestiarum*, and *Pedobacter koreensis* identified (Table S6).

### 16S rRNA gene metabarcoding

Metabarcoding analysis was conducted on 51 biofilm samples (one or two per farm), including 27 from 15 broiler houses and 24 from 13 pig nursery units. After rarefaction analysis, all samples reached a plateau. The reads generated per sample ranged from 55,712 to 283,223, with an average of 126,363. The ratio of bases that have phred quality score of over 20 ranged from 90 to 96%. Furthermore, taxa with fewer than 10 reads were removed. Taxonomy was assigned at the genus level.

#### Bacterial taxonomic abundance analysis

For each farmhouse, the sample with the highest number of sequencing reads was selected and used as the reference in the relative taxonomic abundance analysis. The biofilm bacterial communities in the broiler and pig nursery sectors were dominated by Proteobacteria (relative abundance of 53.6% and 53.7%, respectively), Firmicutes (30.5%, 19.3%), Actinobacteriota (8.6%, 11.6%), and Bacteroidota (4.4%, 8.6%).

**Fig. 5:**
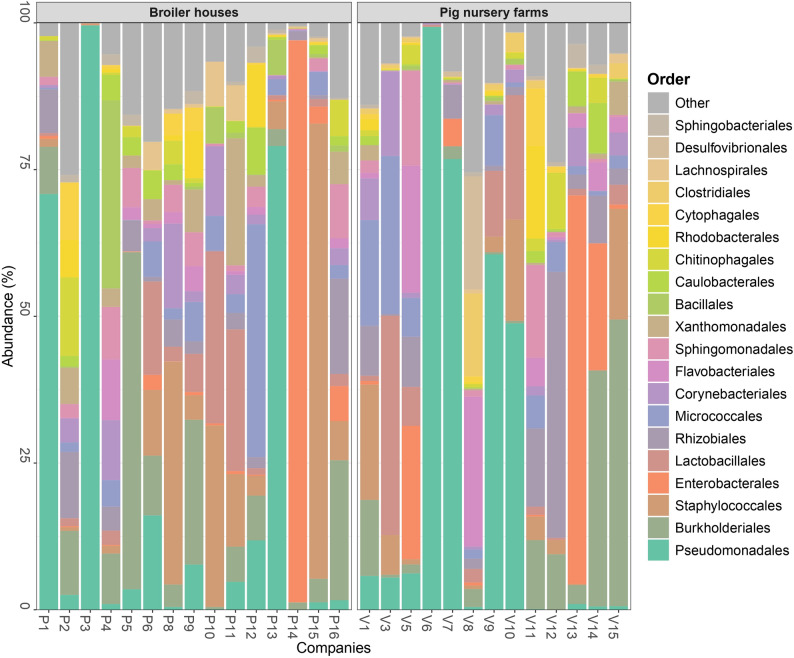
Cumulated histograms representing the relative abundance of taxa identified by metabarcoding at family levels (top 25) from biofilm swabs taken at broiler houses and pig nursery units

At the family level, the most abundant groups in the broiler and pig nursery sectors, respectively, were *Pseudomonadaceae* (12.0%, 10.2%), *Staphylococcacea*e (16.4%, 7.0%), and *Moraxellaceae* (8.7%, 9.1%; Fig. 5). Within these families, the dominant genera were *Pseudomonas* (12.0%, 10.2%) and *Staphylococcus* (15.4%, 5.2%) for the broiler and pig nursery sectors, respectively. *Acinetobacter* (8.6%) was the primary genus within *Moraxellaceae* in broiler houses, whereas *Psychrobacter* (8.9%) was dominant in pig nursery units (data not shown).

Additional prominent families in the broiler sector included *Lactobacillaceae* (6.1%), *Comamonadaceae* (5.9%), *Aeromonadaceae* (5.7%), and *Xanthomonadaceae* (4.3%), with *Lactobacillus* (3.8%), *Hydrogenophaga* (1.3%), *Aeromonas* (5.7%), and *Stenotrophomonas* (3.1%) as the main genera, respectively (data not shown). In the pig nursery sector, notable families were *Enterobacteriaceae* (4.9%), *Comamonadaceae* (4.7%), and *Flavobacteriaceae* (4.5%), with *Citrobacter* (3.1%), *Acidovorax* (1.3%), and *Flavobacterium* (4.4%) as the dominant genera, respectively (Fig. 5).

#### Bacterial alpha diversity

Bacterial alpha diversity was evaluated using the Shannon and Simpson (Gini) indices, with results grouped by farm type, source water, and water treatment (Fig. S1). The analysis revealed no statistically significant differences across farm types, source water, or DWS disinfection (significance at *p* < 0.05). However, some trends were apparent: broiler houses exhibited slightly higher overall diversity according to the Shannon index, with fewer dominant genera compared to the pig sector, as indicated by the Simpson index (Fig. S1A). Also, both sectors had Simpson indices close to one, reflecting fewer dominant genera. Among the different water types, rainwater samples (n = 2) had the highest bacterial diversity and were dominated by fewer genera (Fig. S1B). Bacterial diversity and dominance, as measured by the Shannon and Simpson indices, with diversity ranking from highest to lowest as: rainwater, groundwater, tap water and surface water. Finally, samples from potassium peroxymonosulfate-treated DWS (n = 2) had the highest bacterial diversity, while chlorine-treated DWS had the lowest (Fig. S1C). Diversity and dominance were ranked from highest to lowest as: peroxymonosulfate, hydrogen peroxide, none, chlorine dioxide and chlorine-treated samples.

#### Bacterial beta diversity

**Fig. 6:**
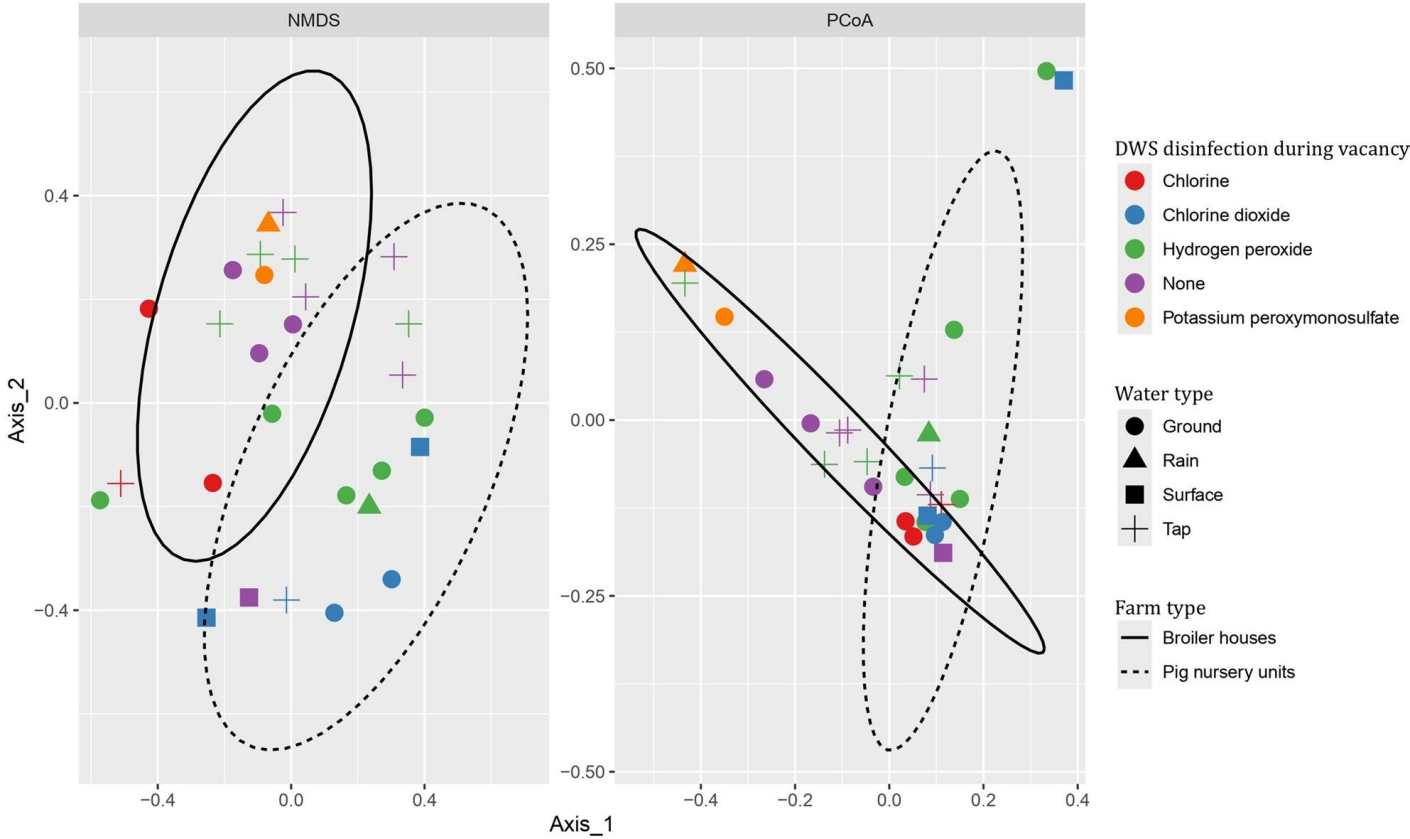
Non-metric multidimensional scaling (NMDS) and principal coordinates analysis (PCoA) plots showing differences in bacterial community composition at the genus level across water samples from different farm types (broiler houses and pig nursery units), source waters, and DWS disinfection. Each point represents a sample, coloured by the type of disinfectant used and shaped by the source water. Confidence ellipses (95%) represent clustering by farm type (solid for broiler houses, dashed for pig nurseries). Clear separation between ellipses suggests distinct microbial communities between farm types, with additional variation related to disinfection method and source water

The bacterial community diversity across different farm types, source waters, and treatments was analysed with NMDS and PCoA (Fig. 6). Both NMDS and PCoA plots revealed a general separation between the two farm types, suggesting differences in bacterial community composition. The assumption of homogeneity of variances was only met for the farm type variable (*p* = 0.3087). A PERMANOVA test was then conducted to further investigate the effect of farm type on bacterial community composition. The test indicated that the farm type explained 5.15% of the total variation (R^2^ = 5.15%) and with a p-value of 0.088, this effect was not statistically significant.

## Discussion

### Contamination in broiler houses and pig nursery units

#### DWS surface contamination

No significant differences were observed in TAC between the two farm types during vacancy before cleaning and disinfection (C&D), suggesting that the conditions facilitating microbial colonisation are broadly similar across broiler houses and pig nursery units, as their DWS, being closed, moist and low-flow water systems, provide an ideal environment for biofilm proliferation. In addition, the recommended temperature during production, 20 °C to 32 °C for broilers and 22 °C to 28 °C for piglets, advised by the Flemish Agency for Agriculture and Fisheries [60, 61], favour microbial proliferation [62]. Our results aligned with previous research, which reported TAC levels in broiler house DWS post-C&D of 4.27 to 7.19 log CFU/20 cm^2^ [25] and slightly higher than in pig abattoir lairage DWS with counts between 0.85 to 4.63 log CFU/cm^2^ [4].

Despite the application of water treatments and disinfectants such as hydrogen peroxide and chlorine dioxide in 21 of the 30 participating farmhouses, microbial loads can be high at the end of the production round with median level of 3.6 log CFU/cm^2^. Besides, faecal contaminants such as enterococci and *E.* coli were detected in 24 farmhouses, which may increase the mortality of the animals by causing gastroenteritis [63, 64]. Furthermore, *C perfringens*, a known pathogen associated with enteritis in piglets [65], was mainly present in pig nursery units. This can be explained by differences in gastrointestinal microbiota and in the design of the DWS [66]. In broiler houses, water lines are typically straight with drinking nipples attached, allowing for a more uniform water flow. In contrast, water lines in pig nursery units often contain numerous bends, which increase sediment accumulation in corners. As *C. perfringens* is anaerobic, these sediments serve as reservoirs [67]. Besides bacterial contaminants, yeasts and moulds were also detected in 26 out of the 30 farmhouses. Previous studies have demonstrated that fungi can form biofilms in DWS, with *Aspergillus*, *Cladosporium*, and *Penicillium* spp. being the most identified genera [68–70]. Moreover, interactions among fungal species, both competitive and synergistic, can influence biofilm development [68, 71].

Farmhouses that did not implement C&D had similar microbial counts, suggesting that current C&D practices are insufficient for effective biofilm removal. Previous research has demonstrated that C&D protocols in broiler houses often fail to eradicate biofilms, which are protected by the EPS they produce [18, 25]. As biofilms mature, they can release planktonic cells into their environment [17]. Therefore, the presence of faecal contaminants and other potential pathogens in DWS biofilms underscores the risk of their dispersion into the passing drinking water.

#### Characterisation of isolates collected from DWS biofilm samples

The dominant bacterial families identified from TAC were similar in both sectors, with *Staphylococcacea*e and *Pseudomonadaceae* being the most prevalent and found at comparable rates, two families, from which species such as *S. epidermidis* and *P. aeruginosa* are known to form biofilms [72]. *Pseudomonadaceae* are common environmental bacteria often found in soil and water, while *Staphylococcacea*e are commonly found on animal/human skin and mucosal surfaces [73, 74], thus playing an important role in contaminating DWS surface biofilms. Among the *Staphylococcacea*e, *S. saprophyticus* was the most prevalent, found in 18 out of the 30 farmhouses. These coagulase-negative staphylococci (CoNS) are commonly associated with urinary tract infections in both humans and animals [75]. Previous studies have shown that CoNS strains isolated from broiler and pig farms, such as *Staphylococcus gallinarum*, *Staphylococcus borealis* and *S. saprophyticus*, often exhibit multidrug resistance [76, 77]. Additionally, CoNS species are known for their ability to form biofilms, which enhances their resistance to antibiotics [78, 79]. One study found that 93% of *S. saprophyticus* isolates from human infections produced biofilms [80]. Within the *Pseudomonadaceae* family, *P. aeruginosa* was most common in broiler houses (7 out of 15 farmhouses), while *P. fluorescens* dominated in pig nursery units (5 out of 15 farmhouses). *P. aeruginosa* was added in 2024 to the World Health Organization's Priority Pathogens [81]. Previous studies have linked *P. aeruginosa* to increased mortality in broiler houses [8, 25, 82]. *P. fluorescens* species are commonly found in diverse environments, including water, soil, and the microbiota of humans and animals [57]. Additionally, these species have demonstrated adaptive biofilm formation under hydrodynamic stress and disinfectant tolerance [21, 83]. Furthermore, studies on dual-species biofilms involving *Staphylococcus* and *Pseudomonas* spp. have revealed accelerated biofilm development and increased tolerance to antibiotics and disinfectants [19, 84, 85]. Since these species were dominant and widely distributed across the farmhouses, further research is essential to address biofilm-related challenges associated with these two genera.

While the two dominant families were consistent, the occurrence of other bacterial families varied. Since the source waters are similar across sectors, differences in bacterial occurrence are more likely explained by structural factors of the DWS and the animals’ microbiota, which vary between species. For example, in broiler houses, the families *Comamonadaceae* and *Xanthomonadaceae* were more frequently identified, including species such as *Pseudacidovorax intermedius*, *Acidovorax delafieldii*, (*Comamonadaceae*) and *Stenotrophomonas maltophilia* (*Xanthomonadaceae*). Previous research on biofilms in DWS has identified similar species, emphasizing their importance in biofilm communities [25, 86]. *S. maltophilia* is an opportunistic and drug-tolerant pathogen that has also been associated with *P. aeruginosa* biofilms and co-infections [25, 87]. In contrast, *Moraxellaceae* were predominant in pig nursery units, with *P. faecalis/pulmonis* frequently identified, species prevalent in the tonsil and respiratory microbiome of pigs that can contaminate DWS surfaces through drinking bowls containing standing water [88–90]. The dominant bacterial species varied across farms and did not always correspond with those most frequently isolated, likely partly due to methodological constraints such as the limited number of colonies (1 to 10) selected from the highest dilution plate. Additionally, variation in microbiota composition may reflect farm-specific conditions, including differences in animal type, source water, nutrient input, ambient temperature, environmental exposure, and C&D protocols.

#### Drinking water contamination and microbial characterization from source to nipple

Participating farmers used various water types as sources for their drinking water (ground, surface, rain and tap), with groundwater being the most common, followed by tap water. Between the source water and the drinking nipple water, typical water treatments such as filtration and acidification were applied. Furthermore, drinking water disinfection (physical or chemical) was used at 21 out of the 30 farmhouses. In the source water, the TAC at both temperatures (22 °C and 36 °C) of the drinking water from the broiler houses was significantly lower than at the drinking nipples. In contrast to this parameter, no significant difference was observed between sampling locations within pig nursery units. Analysing the enumerations of the more specific microbiological parameters did not reveal differences between the source water and drinking nipple water for either sector.

Comparing the results with the microbiological standards of drinking water set by the Animal Health Care Flanders [10, 11], only two pig nursery units did not meet the TAC standards (< 5.0 log CFU/ml), one of each sector did not meet the coliform standards (< 4.0 log CFU/100 ml), and one broiler house didn’t meet the *E. coli* standards (< 3.0 CFU/100 ml). However, with standards set at less than 1 CFU/ml for enterococci and *C. perfringens*, these bacteria were enumerated higher in 11 broiler houses and 9 pig nursery units for enterococci. At the same time, 9 pig nursery units also exceeded the *C. perfringens* requirement, which could potentially lead to animal health problems and affect production efficiency [91]. Although microbiological quality at the source water mostly met the requirements for animal drinking water, a general decline in microbiological quality was observed at the drinking nipples, despite water treatments and disinfection. This trend aligns with results from a previous study on turkey farms [92].

Dominant species, for both sectors, inside the DWS, also found in the source water, were *P. fluorescens* and *S. arlettae* in 6 out of the 30 farmhouses. At the drinking nipple, dominant bacteria identified in the biofilm samples, such as *S. maltophilia, P. fluorescens, P. aeruginosa* and *S. saprophyticus*, were identified in 10 of the 30 farmhouses' drinking water samples (in 8 broiler houses and 2 pig nursery units). Since we only identified the dominant microbiota of the water samples using the TAC plates, some species may not have been isolated. However, dominant species do not present in the source water that were present at the drinking nipple (mainly *P. aeruginosa* and *S. maltophilia* in broiler houses) and the correlation between the surface sample counts and the drinking water bacterial load at the drinking nipple for TAC 37 °C suggests that water contamination could be due to the biofilm present in DWS.

#### Environmental characteristics

The results revealed a negative correlation between the pH of the drinking water at the nipple and the TAC in the corresponding biofilm and water samples, indicating that lower pH levels were associated with higher bacterial biofilm formation. Furthermore, the total hardness of the drinking water at the nipple was positively correlated with the TAC in the corresponding biofilm and water samples. According to the World Health Organization, water with hardness levels exceeding 20°fH (200 mg/L) can lead to scale buildup on pipe surfaces [93]. In this study, 17 farmhouses surpassed this threshold, with an overall median hardness of 31°fH. Hard water contributes to the formation of rough internal pipe surfaces, which can facilitate biofilm adhesion and persistence, and lead to contamination of passing water [92].

### Metabarcoding analysis of the biofilm surface samples

Plating methods have limitations when characterising an entire environmental microbiota, as analyses are constrained to specific culture media and incubation temperatures, and because not all bacteria have been identified. That is why 16S rRNA metabarcoding of the biofilm samples was performed to provide deeper insights into the bacterial community. These results aligned well with the dominant taxa identified from TAC plate isolates, where *Pseudomonadaceae* and *Staphylococcacea*e were the two most abundant families. Metabarcoding analyses also frequently detected *Aeromonadaceae* and *Lactobacillaceae* in broiler houses and *Comamonadaceae* in pig nursery units, which were underrepresented in the TAC-based culture isolates. This difference was explained by the fact that metabarcoding provides a better overview since both culturable and non-culturable cells were detected, as well as anaerobic bacteria or bacteria that do not grow at 21 °C or 37 °C.

Bacterial alpha diversity, assessed using Shannon and Simpson indices, showed no significant differences across farm types, source waters, or DWS disinfections (*p* < 0.05). These results suggest that while there are some variations in microbial communities, they may not be large enough to result in clear, statistically significant differences. The lack of distinct patterns in diversity across water types and treatments indicates that microbial communities in DWS are influenced by a complex interplay of factors rather than a single dominant variable. Moreover, there are only a limited number of samples per variable. A larger number of samples per variable would be needed to make further conclusions.

### Conclusions

This study provides the first comprehensive characterization of biofilm occurrence and microbiota in the DWS of broiler houses and pig nursery units. The findings highlight the complex and diverse nature of microbial contamination in DWS, with possible high bacterial loads detected despite water treatments and disinfection. The frequent presence of known biofilm formers and potential pathogens, including *P. aeruginosa* and faecal indicator organisms, highlights the risk of transmission to animals via drinking water. Across both broiler houses and pig nursery units, *Staphylococcus* and *Pseudomonas* were the most frequently isolated genera in biofilm samples. *S. saprophyticus* species were frequently detected across all farmhouses. In broiler houses, *P. aeruginosa* and *S. maltophilia* were the most prevalent species, whereas in pig nursery units, *P. fluorescens* and *P. faecalis/pulmonis* were the most prevalent.

Water sample analyses showed that microbial contamination was influenced not only by the quality of the source water but also by biofilms on DWS pipeline surfaces. Although several microbial groups were detected in both source water and drinking nipple water, only TAC and coliform counts in broiler houses increased significantly from source water to drinking nipple water. Nevertheless, the detection of similar bacterial taxa in both water and water-contact surfaces demonstrates that biofilms serve as a persistent reservoir of potential pathogens that can contaminate the flowing water. These findings underscore the need for targeted strategies to monitor and reduce biofilm formation within livestock drinking water systems. Our findings form a good basis for future research on microbiological water quality and microbial ecology of biofilms within DWS. Knowledge of bacterial load and characterisation of biofilms in DWS is essential for evaluating the effectiveness of C&D protocols and water treatments in livestock settings. This study also lays the groundwork for upcoming in vitro and in vivo investigations planned by our research group.

## Supplementary Information

Supplementary Material 1.

## Data Availability

Raw metabarcoding data that support the findings of this study have been deposited NCBI Sequence Read Archive (SRA) under the BioProject accession number PRJNA1311316: https://www.ncbi.nlm.nih.gov/bioproject/PRJNA1311316.
