# Supplementary material for: Hidden threats: exploring biofilm communities in broiler houses and pig nursery units drinking water lines
Source: BMC Microbiol. 2026 Mar 5;26:338. doi: 10.1186/s12866-026-04790-6 (PMC13077942; doi:10.1186/s12866-026-04790-6)
Supplement: Supplementary file 1 — Supplementary Material 1. [file 12866_2026_4790_MOESM1_ESM.docx]

**Supplementary material**

**Table S1** Values for total aerobic count (TAC) at 21°C and 37°C, coliforms, enterococcus, yeast and moulds, presumptive C. perfringens, Pseudomonas spp. enumerations and E. coli detection from swab samples collected from the inside of the pipeline of drinking water systems within pig nursery units and broiler houses for each farm.

The number of sampled points (n) for each farm sampling. Samples starting with V originate from pig nursery farms and samples starting with P originate from broiler houses. Mean and standard deviation are given for values that are normally distributed. First quartile (Q1), median (Q2, in bold) and third quartile (Q3) are given for values that did not follow a normal distribution.

**Table S2** Environmental characteristics (pH, Temperature (°C), Total oxidized nitrogen (mg/L), Total orthophosphate (mg/L), Total hardness (F°) and conductivity at 25°C (µS/cm)OS (mg/L)) of the source and drinking nipple water samples taken at broiler houses and pig nursery units

| Sample | Location | pH | T°C | Total Oxidized  Nitrogen (mg/L) | Total Orthophosphate (mg P/L) | Total Hardness (F°) | Conductivity 25°C (μS/cm) |
| --- | --- | --- | --- | --- | --- | --- | --- |
| P1 | Source | 7,4 | 11,8 | 54,0 | 0,079 | 73,6 | 1525 |
|  | Drinking nipple | 7,6 | 19,5 | 49,0 | 0,069 | 37,0 | 1644 |
| P2 | Source | 7,2 | 12 | <0,5 | <0,050 | 38,0 | 744 |
|  | Drinking nipple | 7,2 | 21,5 | <0,5 | <0,050 | 38,3 | 825 |
| P3 | Source | 7,2 | 15,7 | 3,2 | <0,050 | 53,7 | 1070 |
|  | Drinking nipple | 7,2 | 21,2 | 1,4 | <0,050 | 55,1 | 1090 |
| P4 | Source | 8,3 | n.r. | <0,1 | <0,050 | 10,0 | n.r. |
|  | Drinking nipple | 8,2 | n.r. | 0,2 | 0,050 | 8,8 | n.r. |
| P5 | Source | 7,9 | n.r. | <0,10 | <0,050 | 33,0 | n.r. |
|  | Drinking nipple | 9,3 | n.r. | 0,2 | <0,050 | 24,9 | n.r. |
| P6 | Source | 8,1 | n.r. | 0,1 | <0,050 | 16,2 | n.r. |
|  | Drinking nipple | 7,9 | n.r. | <0,1 | <0,050 | 16.2 | n.r. |
| P8 | Source | 7,3 | 15,2 | 31,0 | 0,060 | 65,5 | 1375 |
|  | Drinking nipple | 7,4 | 23,2 | 31,0 | 0,068 | 0,3 | 1490 |
| P9 | Source | 7,6 | n.r. | 3,2 | <0,050 | 37,8 | 787 |
|  | Drinking nipple | 7,6 | n.r. | 3,2 | <0,050 | 37,7 | 792 |
| P10 | Source | 8,2 | n.r. | 0,3 | <0,050 | 15.2 | n.r. |
|  | Drinking nipple | 8,1 | n.r. | 0,3 | <0,050 | 16,3 | n.r. |
| P11 | Source | 7,9 | 10,0 | 5,8 | <0,050 | 31,4 | 1221 |
|  | Drinking nipple | 7,8 | 20,0 | 5,8 | <0,050 | 31,4 | 1225 |
| P12 | Source | 7,4 | 18,6 | 4,9 | <0,050 | 36,9 | 942 |
|  | Drinking nipple | 7,4 | 23,8 | 5,0 | <0,050 | 37,3 | 914 |
| P13 | Source | 7,3 | n.r. | 6,1 | <0,050 | 35,6 | 762 |
|  | Drinking nipple | 7,3 | n.r. | 5,9 | <0,050 | 36,8 | 805 |
| P14 | Source | 8,2 | n.r. | <0,1 | 0,810 | 55,7 | n.r. |
|  | Drinking nipple | 6,7 | n.r. | <0,1 | 0,520 | 59,9 | n.r. |
| P15 | Source | 7,4 | 15,5 | <0,1 | n.r. | 38,6 | n.r. |
|  | Drinking nipple | 7,6 | 17,1 | <0,1 | n.r. | 41,3 | n.r. |
| P16 | Source | 7,8 | 17,7 | <0,5 | <0,050 | 39,5 | 922 |
|  | Drinking nipple | 7,5 | 20,2 | <0,5 | <0,050 | 37,7 | 933 |
| V1 | Source | 7,5 | 9,8 | 5,3 | 0,300 | 28,0 | 808 |
|  | Drinking nipple | 7,4 | 13,1 | <0,5 | 2,180 | 12,0 | 1976 |
| V2 | Source | 7,5 | 7,3 | 7,5 | 0,076 | 17,2 | 635 |
|  | Drinking nipple | 7,7 | 9,8 | 7,7 | 0,076 | 17,5 | 652 |
| V3 | Source | 7,8 | 10,0 | 3,0 | <0,050 | 39,7 | 789 |
|  | Drinking nipple | 7,8 | 10,0 | 2,9 | 0,051 | 31,9 | 710 |
| V4 | Source | 7,3 | 2,9 | 18,0 | 0,200 | 27,7 | 724 |
|  | Drinking nipple | 7 | 9,7 | 14,4 | 0,062 | 26,2 | 823 |
| V5 | Source | 8,1 | 12,3 | 9,6 | <0,050 | 6,8 | 162 |
|  | Drinking nipple | 7,8 | 12,6 | 3,5 | <0,050 | <0,05 | 193 |
| V6 | Source | 8,7 | 8,5 | 1,8 | <0,050 | 13,8 | 488 |
|  | Drinking nipple | 8,3 | 18,9 | 2,0 | 0,058 | 12,8 | 524 |
| V7 | Source | 9,2 | 8,9 | 2,7 | 0,300 | 21,8 | 576 |
|  | Drinking nipple | 9 | 12,8 | 2,8 | 0,400 | 22,4 | 601 |
| V8 | Source | 7,5 | 17,0 | 0,6 | 0,610 | 13,8 | 459 |
|  | Drinking nipple | 3,8 | 18,8 | 0,8 | 0,680 | 13,8 | 645 |
| V9 | Source | 8,1 | 16,0 | <0,5 | 0,330 | 7,5 | 4087 |
|  | Drinking nipple | 8,1 | 17,0 | <0,5 | 0,360 | 7,4 | 4080 |
| V10 | Source | 8,2 | n.r. | <0,1 | 0,810 | 55,7 | n.r. |
|  | Drinking nipple | 6,9 | n.r. | <0,1 | 0,570 | 54,2 | n.r. |
| V11 | Source | 7,7 | 23,1 | 1,5 | 0,106 | 16,7 | 481 |
|  | Drinking nipple | 7,8 | 23,2 | 2,0 | 0,860 | 30,6 | 708 |
| V12 | Source | 7,6 | 20,3 | 3,2 | 0,061 | 39,4 | 839 |
|  | Drinking nipple | 3,6 | 20,3 | 3,2 | 0,081 | 39,6 | 2593 |
| V13 | Source | 8,3 | n.r. | <0,5 | <0,050 | 11,2 | 264 |
|  | Drinking nipple | 8,2 | n.r. | <0,5 | <0,050 | 11,3 | 269 |
| V14 | Source | 7,5 | 19,2 | 26,0 | 0,109 | 43,7 | 1080 |
|  | Drinking nipple | 7,5 | 15,9 | 7,0 | 0,200 | 7,1 | 1018 |
| V15 | Source | 7,4 | 10,0 | 2,6 | 0,200 | 11,4 | 278 |
|  | Drinking nipple | 3,5 | 21,4 | 1,3 | 1,260 | 8,0 | 365 |

n.r. = not reported

**Table S3** Correlation table with Spearman's r and p values (two-tailed) between TAC of drinking water samples and biofilm swabs. Significant values are marked in bold.

|  | TAC 22°C of drinking water  samples at the drinking nipples | TAC 36°C of drinking water  samples at the drinking nipples |
| --- | --- | --- |
| TAC 22°C of drinking water  samples at the source | **0.5487** |  |
|  | **p = 0.0045** |  |
| TAC 36°C of drinking water  samples at the source |  | 0.2847 |
|  |  | p = 0.1880 |
| TAC 21°C of biofilm swabs | 0.2270 |  |
|  | p = 0.2363 |  |
| TAC 37°C of biofilm swabs |  | **0.5195** |
|  |  | **p = 0.0065** |

**Table S4** Correlation table with Spearman's r and p values (two-tailed) between TAC of drinking water samples, biofilm swabs and the environmental characteristics of the drinking water samples taken at the drinking nipples. Significant values are marked in bold.

|  | pH | Total Oxidized  Nitrogen | Total  Orthophosphate | Total Hardness | Conductivity  25°C |
| --- | --- | --- | --- | --- | --- |
| TAC 22°C of drinking water  samples at the drinking nipples | -0.104 | -0.1858 | -0.1239 | 0.1171 | **-0.4591** |
|  | p = 0.5914 | p = 0.4329 | p = 0.6338 | p = 0.5527 | **p = 0.0275** |
| TAC 36°C of drinking water  samples at the drinking nipples | **-0.5447** | 0.2012 | 0.09011 | **0.4646** | -0.1247 |
|  | **p = 0.0040** | p = 0.4233 | p = 0.7616 | **p = 0.0193** | p = 0.5903 |
| TAC 21°C of biofilm swabs | -0.2859 | 0.1107 | 0.001231 | -0.02569 | -0.07794 |
|  | p = 0.1256 | p = 0.6329 | p = 0.9981 | p = 0.8948 | p = 0.7237 |
| TAC 37°C of biofilm swabs | **-0.6239** | 0.1184 | 0.1532 | **0.4312** | 0.03629 |
|  | **p = 0.0002** | p = 0.6094 | p = 0.5542 | **p = 0.0195** | p = 0.8694 |

**Table S5** Genus and species identity of isolates of biofilm samples from PAB, RAPID’E.coli 2 and S&B.

| **Species** | **Broiler houses** | | |  | **Pig nursery units** | | |
| --- | --- | --- | --- | --- | --- | --- | --- |
|  | **PAB** | **RAPID'E.coli 2** | **S&B** |  | **PAB** | **RAPID'E.coli 2** | **S&B** |
|  | **n = 56** | **n = 43** | **n = 28** |  | **n = 29** | **n = 45** | **n = 14** |
| **Gram positive (n = 42)** |  |  |  |  |  |  |  |
| *Staphylococcus arlettae* | 0 | 0 | 18%(5) |  | 0 | 0 | 50%(7) |
| *Staphylococcus saprophyticus* | 0 | 0 | 14%(4) |  | 0 | 0 | 14%(2) |
| *Enterococcus casseliflavus* | 0 | 0 | 14%(4) |  | 0 | 0 | 0 |
| *Enterococcus faecium* | 0 | 0 | 14%(4) |  | 0 | 0 | 0 |
| *Aerococcus viridans* | 0 | 0 | 14%(4) |  | 0 | 0 | 0 |
| *Enterococcus faecalis* | 0 | 0 | 7%(2) |  | 0 | 0 | 7%(1) |
| *Enterococcus avium* | 0 | 0 | 4%(1) |  | 0 | 0 | 21%(3) |
| *Enterococcus hirae* | 0 | 0 | 4%(1) |  | 0 | 0 | 0 |
| *Enterococcus mundtii* | 0 | 0 | 4%(1) |  | 0 | 0 | 0 |
| *Enterococcus saccharolyticus* | 0 | 0 | 4%(1) |  | 0 | 0 | 0 |
| *Lactobacillus johnsonii* | 0 | 0 | 4%(1) |  | 0 | 0 | 0 |
| *Enterococcus canintestini* | 0 | 0 | 0 |  | 0 | 0 | 7%(1) |
| **Gram negative (n = 163)** |  |  |  |  |  |  |  |
| *Escherichia coli* | 0 | 19%(8) | 0 |  | 0 | 16%(7) | 0 |
| *Pseudomonas putida* | 16%(9) | 0 | 0 |  | 3%(1) | 0 | 0 |
| *Pseudomonas fluorescens group* | 14% (8) | 0 | 0 |  | 52%(15) | 0 | 0 |
| *Aeromonas caviae* | 4%(2) | 9%(4) | 0 |  | 0 | 2%(1) | 0 |
| *Pseudomonas spp.* | 9%(5) | 2%(1) | 0 |  | 7%(2) | 0 | 0 |
| *Enterobacter asburiae* | 0 | 9%(4) | 0 |  | 0 | 4%(2) | 0 |
| *Leclercia adecarboxylata* | 0 | 9%(4) | 0 |  | 0 | 4%(2) | 0 |
| *Raoultella ornithinolytica* | 2%(1) | 9%(4) | 0 |  | 0 | 2%(1) | 0 |
| *Pseudacidovorax intermedius* | 9%(5) | 0 | 0 |  | 0 | 0 | 0 |
| *Pseudomonas aeruginosa* | 7%(4) | 7%(3) | 0 |  | 3%(1) | 0 | 0 |
| *Enterobacter cloacae* | 4%(2) | 5%(2) | 0 |  | 0 | 0 | 0 |
| *Escherichia hermannii* | 0 | 5%(2) | 0 |  | 0 | 0 | 0 |
| *Klebsiella oxytoca* | 0 | 5%(2) | 0 |  | 0 | 9%(4) | 0 |
| *Stenotrophomonas maltophilia* | 4%(2) | 5%(2) | 0 |  | 0 | 2%(1) | 0 |
| *Pseudoxanthomonas indica* | 4%(2) | 0 | 0 |  | 0 | 0 | 0 |
| *Acinetobacter lwoffii* | 2%(1) | 0 | 0 |  | 3%(1) | 0 | 0 |
| *Acinetobacter radioresistens* | 2%(1) | 0 | 0 |  | 0 | 0 | 0 |
| *Acinetobacter schindleri* | 2%(1) | 0 | 0 |  | 0 | 0 | 0 |
| *Aeromonas bestiarum* | 2%(1) | 2%(1) | 0 |  | 0 | 0 | 0 |
| *Aeromonas eucrenophila* | 2%(1) | 0 | 0 |  | 3%(1) | 7%(3) | 0 |
| *Enterobacter cancerogenus* | 0 | 2%(1) | 0 |  | 0 | 0 | 0 |
| *Lelliottia amnigena* | 0 | 2%(1) | 0 |  | 0 | 11%(5) | 0 |
| *Ochrobactrum grignonense* | 2%(1) | 0 | 0 |  | 0 | 0 | 0 |
| *Pigmentiphaga spp.* | 2%(1) | 0 | 0 |  | 0 | 0 | 0 |
| *Pseudomonas umsongensis* | 0 | 2%(1) | 0 |  | 0 | 0 | 0 |
| *Serratia fonticola* | 0 | 2%(1) | 0 |  | 0 | 0 | 0 |
| *Variovorax spp.* | 2%(1) | 0 | 0 |  | 0 | 0 | 0 |
| *Klebsiella variicola* | 0 | 2%(1) | 0 |  | 0 | 0 | 0 |
| *Citrobacter freundii* | 0 | 0 | 0 |  | 0 | 9%(4) | 0 |
| *Acinetobacter gandensis* | 0 | 0 | 0 |  | 7%(2) | 0 | 0 |
| *Aeromonas salmonicida* | 0 | 0 | 0 |  | 7%(2) | 2%(1) | 0 |
| *Klebsiella pneumoniae* | 0 | 0 | 0 |  | 0 | 7%(3) | 0 |
| *Aeromonas veronii* | 0 | 0 | 0 |  | 0 | 4%(2) | 0 |
| *Cupriavidus campinensis* | 0 | 0 | 0 |  | 0 | 4%(2) | 0 |
| *Pseudomonas guariconensis* | 0 | 0 | 0 |  | 0 | 4%(2) | 0 |
| *Aeromonas media* | 0 | 0 | 0 |  | 3%(1) | 2%(1) | 0 |
| *Pseudomonas abietaniphila* | 0 | 0 | 0 |  | 3%(1) | 0 | 0 |
| *Pseudomonas antarctica* | 0 | 0 | 0 |  | 3%(1) | 0 | 0 |
| *Pseudomonas frederiksbergensis* | 0 | 0 | 0 |  | 3%(1) | 0 | 0 |
| *Aeromonas hydrophila* | 0 | 0 | 0 |  | 0 | 2%(1) | 0 |
| *Citrobacter amalonaticus* | 0 | 0 | 0 |  | 0 | 2%(1) | 0 |
| *Proteus vulgaris* | 0 | 0 | 0 |  | 0 | 2%(1) | 0 |
| **Yeast (n = 10)** |  |  |  |  |  |  |  |
| *Candida catenulata* | 7%(4) | 0 | 0 |  | 0 | 0 | 0 |
| *Trichosporon japonicum* | 0 | 2%(1) | 0 |  | 0 | 0 | 0 |
| **NA** | 7%(4) | 0 | 0 |  | 0 | 2%(1) | 0 |

The number of identified isolates (n) and not annotated isolates (NA). Pseudomonas Agar Base (PAB) and Slanetz and Bartley medium (S&B).

**Table S6** Genus and species identity of isolates of the drinking water samples from PCA.

| **Species** | **Broiler houses** | |  | **Pig nursery units** | |
| --- | --- | --- | --- | --- | --- |
|  | **Source** | **Drinking nipples** |  | **Source** | **Drinking nipples** |
|  | **n = 25** | **n = 35** |  | **n = 24** | **n = 23** |
| **Gram positive (n = 25)** |  |  |  |  |  |
| *Dermacoccus nishinomiyaensis* | 4%(1) | 0 |  | 0 | 0 |
| *Micrococcus Iuteus* | 4%(1) | 0 |  | 0 | 0 |
| *Microbacterium foliorium* | 4%(1) | 0 |  | 0 | 0 |
| *Pseudarthrobacter oxydans* | 4%(1) | 0 |  | 4%(1) | 0 |
| *Agromyces mediolanus* | 0 | 3%(1) |  | 0 | 0 |
| *Brevibacterium iodinum* | 0 | 3%(1) |  | 0 | 0 |
| *Microbacterium maritypicum* | 0 | 3%(1) |  | 0 | 0 |
| *Microbacterium paraoxydans* | 0 | 3%(1) |  | 0 | 0 |
| *Staphylococcus cohnii* | 0 | 3%(1) |  | 0 | 0 |
| *Staphylococcus sciuri* | 0 | 3%(1) |  | 0 | 0 |
| *Bacillus licheniformis* | 0 | 0 |  | 8%(2) | 9%(2) |
| *Bacillus pumilus* | 0 | 0 |  | 0 | 9%(2) |
| *Peribacillus muralis* | 0 | 0 |  | 0 | 9%(2) |
| *Staphylococcus arlettae* | 0 | 0 |  | 8%(2) | 4%(1) |
| *Kocuria palustris* | 0 | 0 |  | 4%(1) | 0 |
| *Microbacterium oxydans* | 0 | 0 |  | 4%(1) | 0 |
| *Nocardia salmonicida* | 0 | 0 |  | 4%(1) | 0 |
| **Gram negative (n = 71 )** |  |  |  |  |  |
| *Stenotrophomonas maltophilia* | 0 | 20%(7) |  | 0 | 0 |
| *Pseudomonas fluorescens group* | 16%(4) | 3%(1) |  | 8%(2) | 0 |
| *Pseudomonas aeruginosa* | 0 | 14%(5) |  | 0 | 0 |
| *Pseudomonas putida* | 4%(1) | 9%(3) |  | 0 | 0 |
| *Aeromonas veronii* | 8%(2) | 0 |  | 0 | 0 |
| *Acidovorax facilis* | 4%(1) | 3%(1) |  | 0 | 4%(1) |
| *Acidovorax temperans* | 4%(1) | 0 |  | 0 | 0 |
| *Aeromonas encheleia* | 4%(1) | 0 |  | 0 | 0 |
| *Alcaligenes faecalis* | 4%(1) | 0 |  | 0 | 0 |
| *Brevundimonas diminuta* | 4%(1) | 0 |  | 4%(1) | 0 |
| *Cellulosimicrobium cellulans* | 4%(1) | 0 |  | 0 | 0 |
| *Pseudomonas umsongensis* | 4%(1) | 0 |  | 0 | 0 |
| *Psychrobacter maritimus* | 4%(1) | 0 |  | 0 | 0 |
| *Sphingobium limneticum* | 4%(1) | 0 |  | 0 | 0 |
| *Acidovorax delafieldii* | 0 | 3%(1) |  | 0 | 0 |
| *Acinetobacter tandoii* | 0 | 3%(1) |  | 0 | 0 |
| *Aeromonas media* | 0 | 3%(1) |  | 0 | 0 |
| *Brevundimonas intermedia* | 0 | 3%(1) |  | 0 | 0 |
| *Chryseobacterium aquaticum* | 0 | 3%(1) |  | 0 | 4%(1) |
| *Delftia acidovorans* | 0 | 3%(1) |  | 0 | 0 |
| *Ensifer adhaerens* | 0 | 3%(1) |  | 0 | 0 |
| *Leucobacter alluvii* | 0 | 3%(1) |  | 0 | 0 |
| *Pseudomonas spp.* | 0 | 3%(1) |  | 0 | 0 |
| *Sphingobacterium multivorum* | 0 | 3%(1) |  | 0 | 0 |
| *Variovorax spp.* | 0 | 3%(1) |  | 0 | 0 |
| *Janithinobacterium lividum* | 0 | 0 |  | 13%(3) | 0 |
| *Aeromonas bestiarum* | 0 | 0 |  | 0 | 9%(2) |
| *Pedobacter koreensis* | 0 | 0 |  | 0 | 9%(2) |
| *Serratia fonticola* | 0 | 0 |  | 8%(2) | 0 |
| *Sphingobacterium faecium* | 0 | 0 |  | 8%(2) | 0 |
| *Chryseobacterium scophthalmum* | 0 | 0 |  | 0 | 4%(1) |
| *Chryseobacterium spp.* | 0 | 0 |  | 4%(1) | 0 |
| *Flavobacterium sinopsychrotolerans* | 0 | 0 |  | 0 | 4%(1) |
| *Flavobacterium succinicans* | 0 | 0 |  | 0 | 4%(1) |
| *Lelliottia amnigena* | 0 | 0 |  | 4%(1) | 4%(1) |
| *Psychrobacter faecalis* | 0 | 0 |  | 0 | 4%(1) |
| *Rhodococcus fascians* | 0 | 0 |  | 4%(1) | 0 |
| *Serratia liquefaciens* | 0 | 0 |  | 0 | 4%(1) |
| *Siccibacter turicensis* | 0 | 0 |  | 4%(1) | 0 |
| *Sphingobium xenophagum* | 0 | 0 |  | 0 | 4%(1) |
| **Yeast (n = 11)** |  |  |  |  |  |
| *Candida tropicalis* | 0 | 0 |  | 0 | 4%(1) |
| *Cyberlindnera jadinii* | 0 | 0 |  | 4%(1) | 4%(1) |
| *Rhodotorula mucilaginosa* | 4%(1) | 0 |  | 0 | 0 |
| *Yarrowia lipolytica* | 0 | 0 |  | 0 | 4%(1) |
| **NA** | 16%(4) | 3%(1) |  | 4%(1) | 0 |

The number of identified isolates (n) and not annotated isolates (NA).

**Fig. S1** Alpha diversity indices (Shannon and Simpson). Box plots of alpha diversity values distribution for all indices (richness and Shannon diversity) of **A**. farm type (broiler houses and pig nursery farms), **B.** water type taken at the drinking nipples (ground, rain, surface and tap) and **C.** water disinfection during vacancy (Chlorin, chlorine dioxide, none, peroxide and sodium hypochlorite) samples are shown. The line inside each box represents the median value. Outliers are shown as dots.

**A.**

**B.**

**C.**


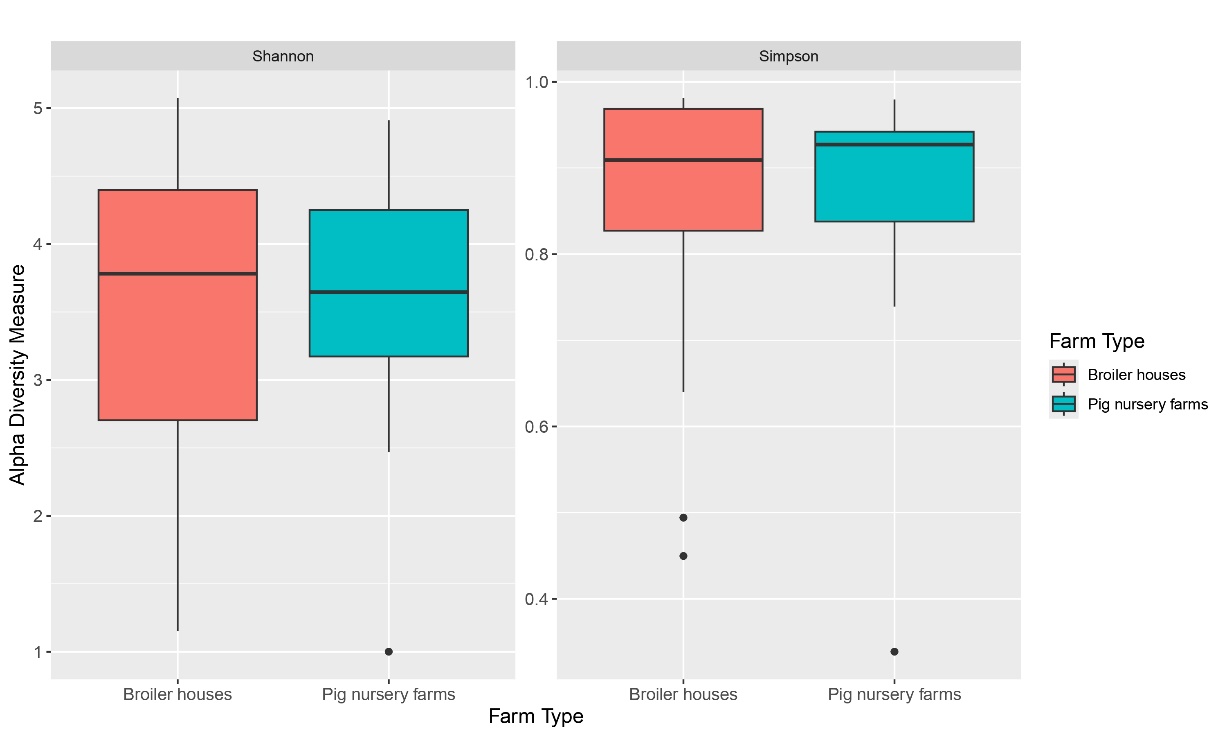

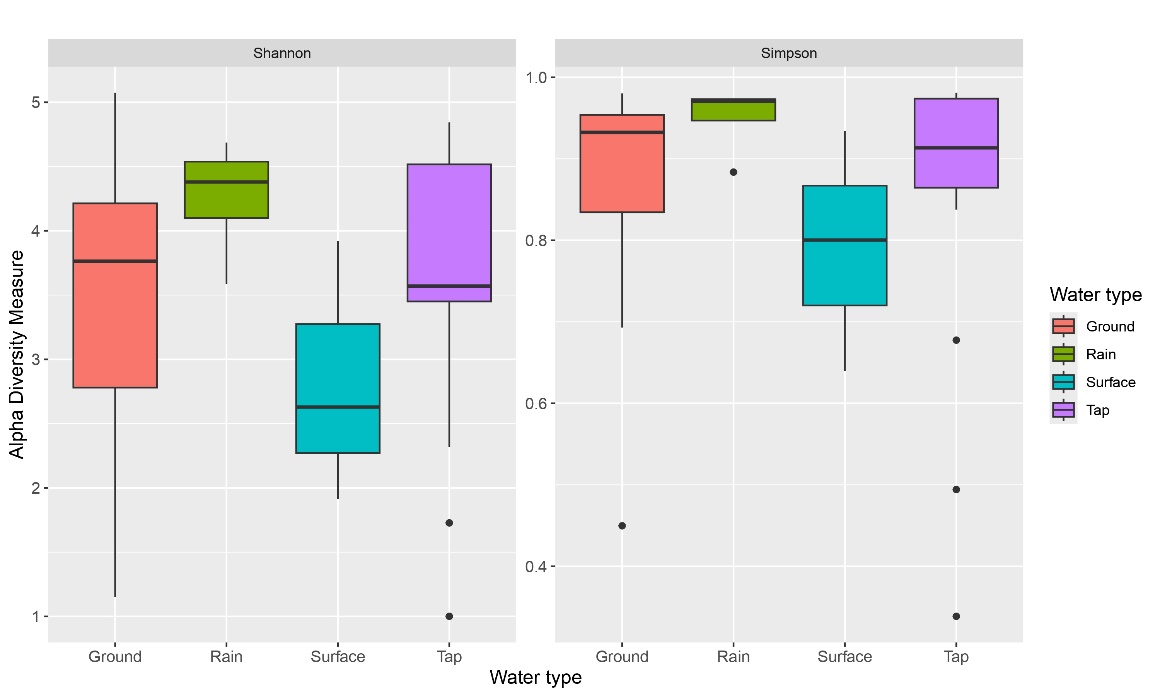

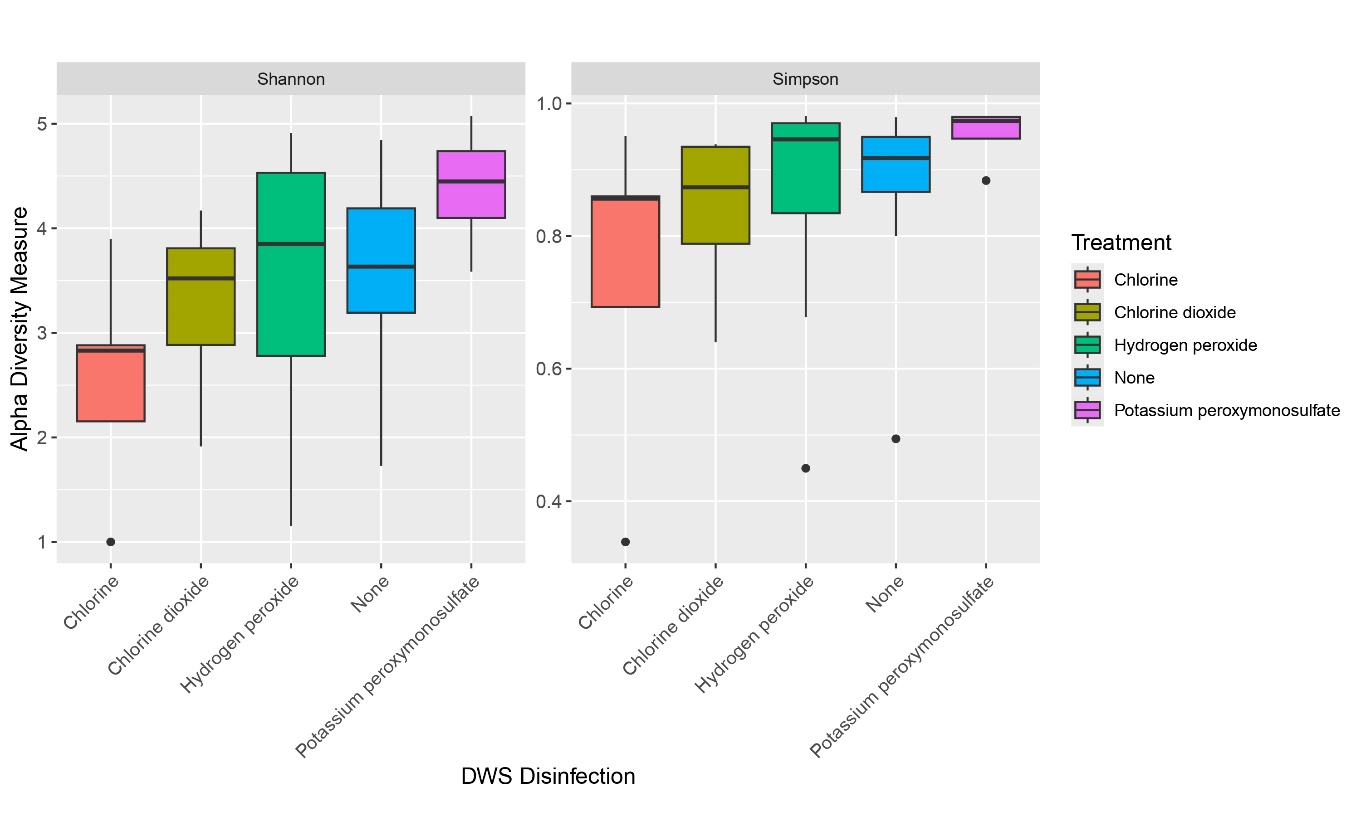


Farm type

Water type

Water disinfection during vacancy
